# Prognostic Significance of Pre- to Postoperative Dynamics of Sarcopenia for Patients with Renal Cell Carcinoma Undergoing Laparoscopic Nephrectomy

**DOI:** 10.3389/fsurg.2022.871731

**Published:** 2022-04-21

**Authors:** Shuqiu Chen, Ting He, Si Sun, Jianping Wu, Bin Xu, Weipu Mao, Ming Chen

**Affiliations:** Department of Urology, Affiliated Zhongda Hospital of Southeast University, Nanjing, China

**Keywords:** renal cell carcinoma, sarcopenia, skeletal muscle index dynamics, prognostic indicator, nephrectomy

## Abstract

**Background:**

The aim of this study was to investigate the prognostic role of the dynamics of sarcopenia in the pre- to postoperative for patients with renal cell carcinoma (RCC) undergoing laparoscopic nephrectomy.

**Methods:**

This study included 261 patients who underwent laparoscopic nephrectomy between 2014 and 2019. The skeletal muscle index (SMI) of the L3 lumbar region was used to assess sarcopenia. The overall population was divided into four groups according to the dynamics of sarcopenia from pre- to postoperative: group 1 (both pre- and postoperative sarcopenia), group 2 (preoperative non-sarcopenia to postoperative sarcopenia), group 3 (preoperative sarcopenia to postoperative non-sarcopenia), and group 4 (both pre- and postoperative non-sarcopenia). The endpoints of the study were overall survival (OS) and cancer-specific survival (CSS).

**Results:**

Of the 261 patients who underwent laparoscopic nephrectomy, 103 (39.5%) had preoperative sarcopenia and 183 (70.1%) had postoperative sarcopenia. Patients with pre- or postoperative sarcopenia had poor survival outcomes. Sarcopenia dynamic was a better predictor of OS (AUC = 0.737) and CSS (AUC = 0.696) in patients with RCC than pre- and postoperative sarcopenia, and patients in group 4 of sarcopenia dynamic had the best OS and CSS. In addition, sarcopenia dynamics was an independent risk factor for OS and CSS, with a 94.5% reduction in OS risk (HR = 0.055, 95% CI 0.007–0.407, *p* = 0.003) and a 91.9% reduction in CSS risk (HR = 0.081, 95% CI 0.011–0.616, *p* = 0.015) in the group 4 compared with the group 1.

**Conclusion:**

Our study is the first to assess the prognostic value of pre- and postoperative sarcopenia dynamics in patients with RCC.

## Introduction

Renal cell carcinoma (RCC), a cancer originating from the parenchymal epithelium of the kidney, accounts for more than 90% of kidney cancer cases (1), and its incidence and mortality rates are gradually increasing at a rate of 2%–3% every 10 years worldwide (2, 3). Despite the increasing rate of early diagnosis of RCC, approximately 20%–30% of patients are diagnosed at an intermediate to advanced stage; even after surgical resection, 20% of patients will still have recurrent metastases during follow-up (4). Although surgical treatment including radical nephrectomy or partial nephrectomy is an effective treatment for early-stage RCC without metastasis, the treatment of advanced RCC remains a great challenge due to the lack of reliable biomarkers associated with RCC (5, 6). Therefore, early identification of prognostic factors in patients with RCC is crucial to improve the survival time of patients.

Malnutrition and frailty are common problems in patients with malignant tumors, and many tumor patients inevitably experience weight loss, which has a negative impact on tumor treatment and prognosis (7, 8). Sarcopenia is a progressive and prevalent skeletal muscle disorder associated with increased likelihood of adverse outcomes including falls, fractures, physical disability and mortality (9, 10). In 2010, the European Working Group on Sarcopenia in Older People (EWGSOP) defined sarcopenia as a syndrome characterized by a progressive and widespread loss of skeletal muscle mass and strength, with a consequent increased risk of adverse outcomes such as physical disability, reduced quality of life, and death (11, 12). Skeletal muscle index (SMI) of the L3 axial plane measured by computed tomography (CT) is a common diagnostic criterion for sarcopenia (13).

A growing number of studies have shown that sarcopenia is associated with poor prognosis in oncology patients and is even an independent risk factor for prognosis (14, 15). Our previous study found sarcopenia assessed by preoperative SMI measurements to be an independent risk factor for RCC patients (16, 17). However, muscle mass, nutrition and inflammatory status may differ preoperatively and postoperatively due to primary tumor eradication, and major surgery can affect the physical condition of patients (18). For this reason, we investigated in the present study the pre- and postoperative sarcopenia dynamics in patients undergoing laparoscopic nephrectomy and assessed the prognostic value of sarcopenia dynamics in patients with RCC.

## Materials and Methods

### Study Design and Patients

**Figure 1:**
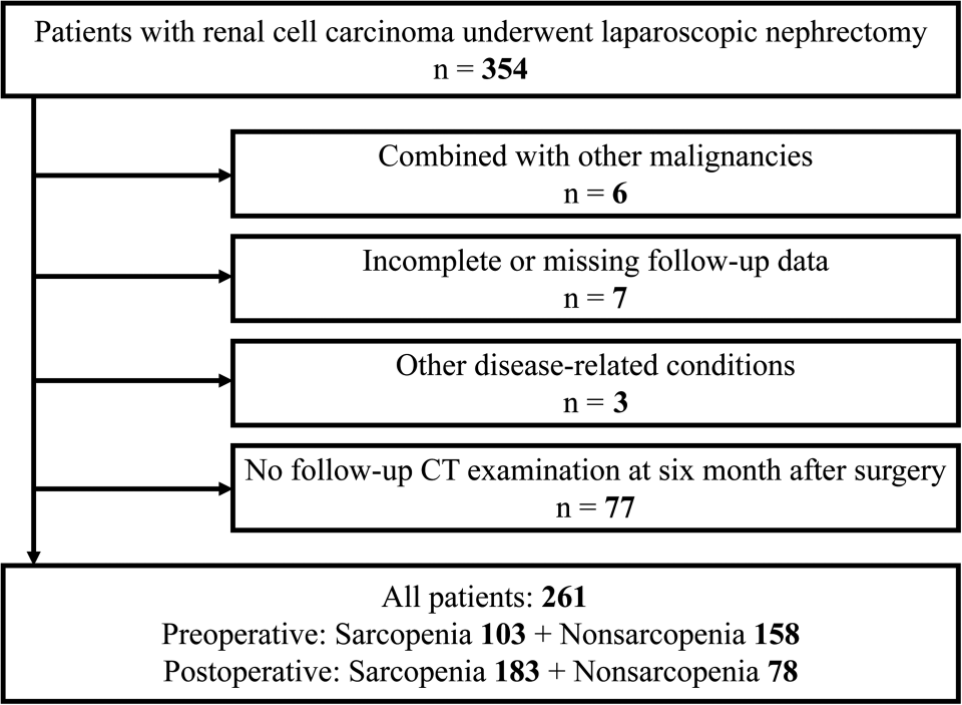
Schematic flow diagram for our study cohort.

We retrospectively collected medical records of 354 patients with RCC who underwent partial or radical laparoscopic nephrectomy at the Department of Urology, Zhongda Hospital Southeast University, from January 2014 to December 2019. Exclusion criteria: six patients with comorbid other malignancies; seven patients with incomplete information on variables or missing follow-up data; three patients with other diseases that affected survival time, and 77 patients who did not review CT examinations six months after surgery (Figure 1). A final total of 261 patients were included. This study was approved by the Ethics Committees and Institutional Review Board of Zhongda Hospital Southeast University (ZDKYSB077).

### Clinical Data Collection and Follow-up

Clinicopathological variables were obtained from hospital electronic medical records, including sex (male and female), age categorized (≤65 years and >65 years), body mass index (BMI, <25 kg/m^2^ and ≥25 kg/m^2^), cardiovascular disease (no and yes), hypertension (no and yes), diabetes (no and yes), smoking (no and yes), type of surgery (partial nephrectomy and radical nephrectomy), AJCC stage (I, II, III and IV), TNM stage, Fuhrman grade (I, II, III and IV), urea nitrogen, creatinine, uric acid, hemoglobin, platelet count, neutrophil count, lymphocyte count, NLR, and PLR. NLR and PLR were calculated as neutrophil number/lymphocyte number, and platelet count/lymphocyte count, respectively. TNM stage of surgical specimens were pathologically classified according to the American Joint Committee on Cancer tumor-node-metastasis (TNM) system. All patients in the study have been followed up by professional staff until December 2020. Urea nitrogen, creatinine, uric acid, hemoglobin, platelet count, neutrophil count, lymphocyte count laboratory test data were obtained two days prior to surgery or closest to the time of surgery.

The primary endpoint of interest in this study was overall survival (OS) after nephrectomy and the secondary endpoint was cancer-specific survival (CSS). SMI was the total muscle area of the psoas, external oblique, internal oblique, paraspinal, rectus abdominis and transverse abdominis muscles on both sides of the level of the L3 vertebrae then normalized to height^2^. Sarcopenia diagnosed according to SMI was determined with reference to our previous studies (16). In the current study, sarcopenia was assessed as more than six months preoperatively and more than six months postoperatively. We divided patients into four different groups based on pre- and postoperative sarcopenia dynamics: group 1 (both pre- and postoperative sarcopenia), group 2 (preoperative non-sarcopenia to postoperative sarcopenia), group 3 (preoperative sarcopenia to postoperative non-sarcopenia), and group 4 (both pre- and postoperative non-sarcopenia).

### Statistical Analysis

Descriptive analysis was performed on clinicopathological variables of patients, and data were expressed as mean, standard deviation, or by frequency of relevant events. To determine statistical differences between groups, we performed the χ^2^ test for categorical variables and the Kruskal-Wallis test for continuous variables. Kaplan-Meier survival curve analysis was used to analyze the survival outcomes of different pre- and postoperative SMI groups and different sarcopenia dynamic groups. Receiver operating characteristic (ROC) curves were used to compare the effects of different pre- and postoperative SMI and different sarcopenia dynamic on predictive ability of OS and CSS, and the results were shown as the area under the curve (AUC). Multivariate Cox regression analysis was used to determine independent risk factors for OS and CSS. Variables that were significant in univariate Cox regression analysis were included in multivariate Cox regression analysis, and the results were described by hazard ratios (HR) and 95% confidence intervals (CI). All statistical analyses for this study were performed using the SPSS statistical program (version 20.0) as well as the R statistical program (version 3.5.3). A two-tailed *P*-value <0.05 was considered statistically significant.

## Results

**Figure 2:**
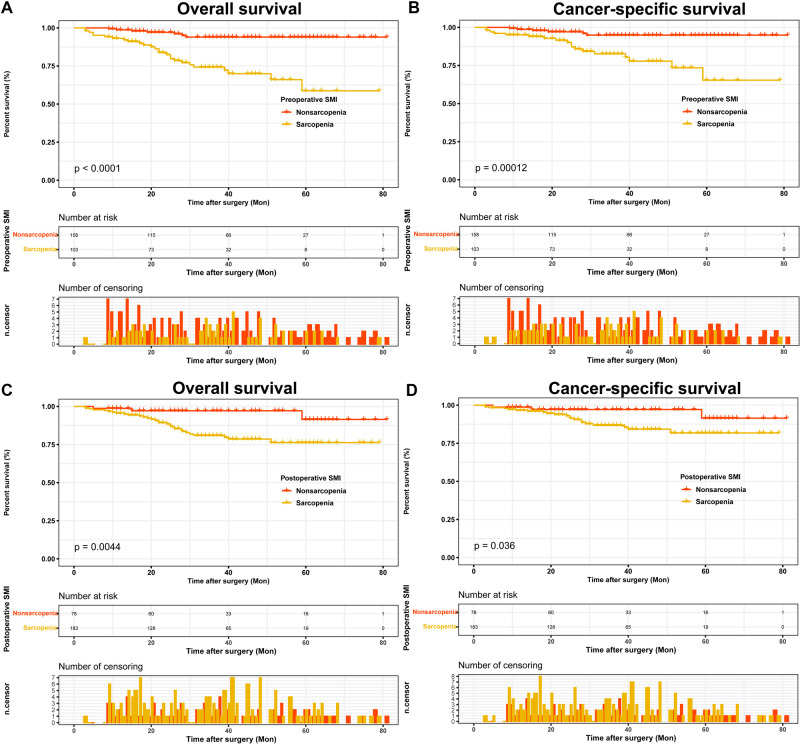
Kaplan-Meier curves for OS and CSS stratified by pre- and postoperative SMI. (**A,B**) Preoperative SMI OS and CSS; (**C,D**) Postoperative SMI OS and CSS. Abbreviations: OS, overall survival; CSS, cancer-specific survival; SMI, skeletal muscle index.

In the entire cohort, 103 (39.5%) patients had sarcopenia as assessed by preoperative SMI and 183 (70.1%) patients had sarcopenia as assessed by postoperative SMI. The clinicopathological characteristics of all patients are shown in Table 1. We found that with or without preoperative sarcopenia was associated with age, type of surgery, AJCC stage, T stage, N stage, M stage and Fuhrman grade, whereas with or without postoperative sarcopenia was associated with age, sex and type of surgery. Both pre- and postoperatively, patients who were older (age >65 years), female and underwent radical nephrectomy were more likely to be evaluated for sarcopenia than patients who were younger (age ≤65 years), male and underwent partial nephrectomy. In addition, Kaplan-Meier curves showed that pre- and postoperative sarcopenia correlated with patients’ OS (preoperative SMI, *p* < 0.0001; postoperative, *p* = 0.0044) and CSS (preoperative SMI, *p* = 0.00012; postoperative, *p* = 0.036), and patients with pre- or postoperative sarcopenia had poor OS and CSS (Figure 2).

**Table 1:** Clinical characteristics of the patients according to pre- and postoperative SMI.

Characteristic	All	Preoperative SMI		*P* value	Postoperative SMI		*P* value
	patients	Nonsarcopenia	Sarcopenia		Nonsarcopenia	Sarcopenia	
	*N* = 261	*N* = 158	*N* = 103		*N* = 78	*N* = 183	
Age categorized, y				0.002			<0.001
≤65	197 (75.5)	130 (82.3)	67 (65.0)		70 (89.7)	127 (69.4)	
>65	64 (24.5)	28 (17.7)	36 (35.0)		8 (10.3)	56 (30.6)	
Sex				0.541			0.023
Male	178 (68.2)	110 (69.6)	68 (66.0)		61 (78.2)	117 (63.9)	
Female	83 (31.8)	48 (30.4)	35 (34.0)		17 (21.8)	66 (36.1)	
BMI categorized, kg/m^2^				0.771			0.676
<25	139 (53.3)	83 (52.5)	56 (54.4)		40 (51.3)	99 (54.1)	
≥25	122 (46.7)	75 (47.5)	47 (45.6)		38 (48.7)	84 (45.9)	
Hypertension				0.479			0.525
No	145 (55.6)	85 (53.8)	60 (58.3)		41 (52.6)	104 (56.8)	
Yes	116 (44.4)	73 (46.2)	43 (41.7)		37 (47.4)	79 (43.2)	
Diabetes				0.725			0.433
No	218 (83.5)	133 (84.2)	85 (82.5)		63 (80.8)	155 (84.7)	
Yes	43 (16.5)	25 (15.8)	18 (17.5)		15 (19.2)	28 (15.3)	
Cardiovascular diseases				0.360			0.142
No	229 (87.7)	141 (89.2)	88 (85.4)		72 (92.3)	157 (85.8)	
Yes	32 (12.3)	17 (10.8)	15 (14.6)		6 (7.7)	26 (14.2)	
Smoking				0.373			0.504
No	217 (83.1)	134 (84.8)	83 (80.6)		63 (80.8)	154 (84.2)	
Yes	44 (16.9)	24 (15.2)	20 (19.4)		15 (19.2)	29 (15.8)	
Surgery type				<0.001			<0.001
Partial nephrectomy	146 (55.9)	109 (69.0)	37 (35.9)		57 (73.1)	89 (48.6)	
Radical nephrectomy	115 (44.1)	49 (31.0)	66 (64.1)		21 (26.9)	94 (51.4)	
Laterality				0.151			0.238
Right	125 (47.9)	70 (44.3)	55 (53.4)		33 (42.3)	92 (50.3)	
Left	136 (52.1)	88 (55.7)	48 (46.6)		45 (57.7)	91 (49.7)	
AJCC stage				0.007			0.834
I	198 (75.9)	129 (81.6)	69 (67.0)		60 (76.9)	138 (75.4)	
II	16 (6.1)	11 (7.0)	5 (4.9)		4 (5.1)	12 (6.6)	
III	33 (12.6)	13 (8.2)	20 (19.4)		11 (14.1)	22 (12.0)	
IV	14 (5.4)	5 (3.2)	9 (8.7)		3 (3.8)	11 (6.0)	
T-stage				0.037			0.890
T1	201 (77.0)	129 (81.6)	72 (69.9)		62 (79.5)	139 (76.0)	
T2	18 (6.9)	12 (7.6)	6 (5.8)		4 (5.1)	14 (7.7)	
T3	35 (13.4)	14 (8.9)	21 (20.4)		10 (12.8)	25 (13.7)	
T4	7 (2.7)	3 (1.9)	4 (3.9)		2 (2.6)	5 (2.7)	
N-stage				0.007			0.486
N0	251 (96.2)	156 (98.7)	95 (92.2)		76 (97.4)	175 (95.6)	
N1	10 (3.8)	2 (1.3)	8 (7.8)		2 (2.6)	8 (4.4)	
M-stage				0.017			0.609
M0	252 (96.6)	156 (98.7)	96 (93.2)		76 (97.4)	176 (96.2)	
M1	9 (3.4)	2 (1.3)	7 (6.8)		2 (2.6)	7 (3.8)	
Fuhrman grade				0.005			0.552
I	41 (15.7)	31 (19.6)	10 (9.7)		15 (19.2)	26 (14.2)	
II	167 (64.0)	103 (65.2)	64 (62.1)		46 (59.0)	121 (66.1)	
III	49 (18.8)	24 (15.2)	25 (24.3)		15 (19.2)	34 (18.6)	
IV	4 (1.5)	0 (0.0)	4(3.9)		2 (2.6)	2 (1.1)	
Urea nitrogen, mmol/L (mean, SD)	6.65, 5.23	6.49, 6.23	6.89, 3.15	0.550	7.29, 8.45	6.37, 2.94	0.194
Creatinine, µmol/L (mean, SD)	114.43, 66.97	112.31, 68.72	117.67, 64.38	0.528	113.49, 73.50	114.83, 64.19	0.883
Uric acid, µmol/L (mean, SD)	254.87, 94.80	251.81, 87.20	259.57, 105.66	0.519	264.32, 87.55	250.85, 97.67	0.294
Hemoglobin, g/L (mean, SD)	134.22, 19.97	133.84, 20.33	134.82, 19.50	0.699	135.06, 17.44	133.86, 21.00	0.657
Platelet Count, 10^9^/L (mean, SD)	219.92, 68.60	221.32, 66.51	217.77, 71.98	0.684	226.50, 62.95	217.11, 70.85	0.312
Neutrophil Count, 10^9^/L (mean, SD)	4.20, 1.62	4.17, 1.62	4.23, 1.62	0.788	4.11, 1.67	4.23, 1.60	0.562
Lymphocyte Count, 10^9^/L (mean, SD)	1.68, 0.53	1.68, 0.50	1.67, 0.57	0.808	1.71, 0.55	1.66, 0.52	0.517
PLR (mean, SD)	141.84, 57.67	141.39, 56.23	142.53, 60.10	0.876	145.38, 59.44	140.33, 57.00	0.518
NLR (mean, SD)	2.77, 1.57	2.76, 1.64	2.79, 1.46	0.873	2.71, 1.61	2.80, 1.55	0.647
Survival months (months)	34.17, 18.90	35.82, 19.77	31.64, 17.25	0.081	37.72, 20.65	32.66, 17.95	0.048

*SMI, skeletal muscle index; BMI, Body mass index; AJCC, American Joint Committee on Cancer.*

**Figure 3:**
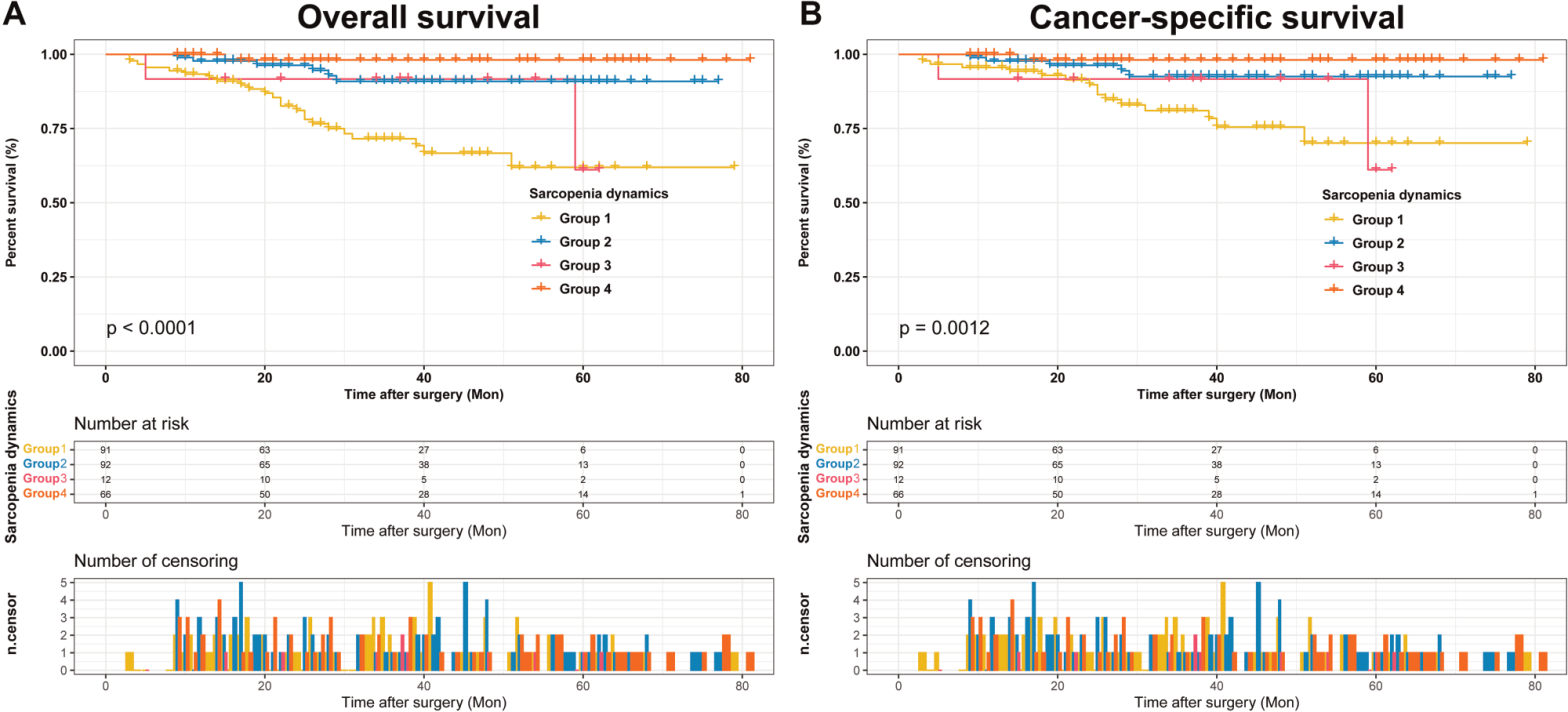
Kaplan-Meier curves for OS and CSS stratified by sarcopenia dynamics. (**A**) Sarcopenia dynamics OS; (**B**) Sarcopenia dynamics CSS. Abbreviations: OS, overall survival; CSS, cancer-specific survival.

Based on the dynamics of pre- and postoperative sarcopenia, 91 (34.9%) patients were included in group 1, 92 (35.2%) patients were classified in group 2, 12 (4.6%) patients were classified in group 3, and 66 (25.3%) patients were divided into group 4 (Table 2). We found that sarcopenia dynamics was associated with age, type of surgery, AJCC stage, T stage, N stage and Fuhrman grade. The Kaplan-Meier curves showed that patients in group 4 had the highest OS and CSS (Figure 3).

**Table 2:** Baseline characteristics of the patients according to sarcopenia dynamics.

Characteristic	Sarcopenia dynamics				*P* value
	Group 1	Group 2	Group 3	Group 4	
	*N* = 91	*N* = 92	*N* = 12	*N* = 66	
Age categorized, y					0.001
≤65	57 (62.6)	70 (76.1)	10 (83.3)	60 (90.9)	
>65	34 (37.4)	22 (23.9)	2 (16.7)	6 (9.1)	
Sex					0.150
Male	58 (63.7)	59 (64.1)	10 (83.3)	51 (77.3)	
Female	33 (36.3)	33 (35.9)	2 (16.7)	15 (22.7)	
BMI categorized, kg/m^2^					0.443
<25	52 (57.1)	47 (51.1)	4 (33.3)	36 (54.5)	
≥25	39 (42.9)	45 (48.9)	8 (66.7)	30 (45.5)	
Hypertension					0.825
No	54 (59.3)	50 (54.3)	6 (50.0)	35 (53.0)	
Yes	37 (40.7)	42 (45.7)	6 (50.0)	31 (47.0)	
Diabetes					0.713
No	75 (82.4)	80 (87.0)	10 (83.3)	53 (80.3)	
Yes	16 (17.6)	12 (13.0)	2 (16.7)	13 (19.7)	
Cardiovascular diseases					0.359
No	78 (85.7)	79 (85.9)	10 (83.3)	62 (93.9)	
Yes	13 (14.3)	13 (14.1)	2 (16.7)	4 (6.1)	
Smoking					0.416
No	75 (82.4)	79 (85.9)	8 (66.7)	55 (83.3)	
Yes	16 (17.6)	13 (14.1)	4 (33.3)	11 (16.7)	
Surgery type					<0.001
Partial nephrectomy	33 (36.3)	56 (60.9)	4 (33.3)	53 (80.3)	
Radical nephrectomy	58 (63.7)	36 (39.1)	8 (66.7)	13 (19.7)	
Laterality					0.395
Right	50 (54.9)	42 (45.7)	5 (41.7)	28 (42.4)	
Left	41 (45.1)	50 (54.3)	7 (58.3)	38 (57.6)	
AJCC stage					0.006
I	65 (71.4)	73 (79.3)	4 (33.3)	56 (84.8)	
II	4 (4.4)	8 (8.7)	1 (8.3)	3 (4.5)	
III	15 (16.5)	7 (7.6)	5 (41.7)	6 (9.1)	
IV	7 (7.7)	4 (4.3)	2 (16.7)	1 (1.5)	
T-stage					0.012
T1	66 (72.5)	73 (79.3)	6 (50.0)	56 (84.8)	
T2	5 (5.5)	9 (9.8)	1 (8.3)	3 (4.5)	
T3	18 (19.8)	7 (7.6)	3 (25.0)	7 (10.6)	
T4	2 (2.2)	3 (3.3)	2 (16.7)	0 (0.0)	
N-stage					0.014
N0	85 (93.4)	90 (97.8)	10 (83.3)	66 (100.0)	
N1	6 (6.6)	2 (2.2)	2 (16.7)	0 (0.0)	
M-stage					0.119
M0	85 (93.4)	91 (98.9)	11 (91.7)	65 (98.5)	
M1	6 (6.6)	1 (1.1)	1 (8.3)	1 (1.5)	
Fuhrman grade					0.001
I	9 (9.9)	17 (18.5)	1 (8.3)	14 (21.2)	
II	58 (63.7)	63 (68.5)	6 (50.0)	40 (60.6)	
III	22 (24.2)	12 (13.0)	3 (25.0)	12 (18.2)	
IV	2 (2.2)	0 (0.0)	2 (16.7)	0 (0.0)	
Urea nitrogen, mmol/L (mean, SD)	6.85, 3.15	5.90, 2.64	7.20, 3.29	7.31, 9.10	0.443
Creatinine, µmol/L (mean, SD)	115.38, 59.59	114.27, 68.77	135.00, 94.91	109.58, 69.09	0.705
Uric acid, µmol/L (mean, SD)	260.13, 105.65	241.66, 88.71	255.33, 110.31	265.95, 83.68	0.581
Hemoglobin, g/L (mean, SD)	133.59, 19.43	134.13, 22.54	144.08, 18.16	133.42, 16.93	0.853
Platelet Count, 10^9^/L (mean, SD)	213.63, 73.00	220.55, 68.89	249.17, 56.76	222.38, 63.54	0.336
Neutrophil Count, 10^9^/L (mean, SD)	4.11, 1.48	4.36, 1.71	5.17, 2.31	3.91, 1.47	0.571
Lymphocyte Count, 10^9^/L (mean, SD)	1.67, 0.57	1.66, 0.47	1.66, 0.62	1.71, 0.54	0.543
PLR (mean, SD)	139.24, 58.28	141.41, 56.01	167.51, 70.25	141.36, 56.95	0.659
NLR (mean, SD)	2.69, 1.33	2.91, 1.75	3.55, 2.17	2.55, 1.45	0.670
Survival months (months)	30.64, 16.95	34.66, 18.76	39.25, 18.42	37.44, 21.14	0.022

*BMI, Body mass index; AJCC, American Joint Committee on Cancer.*

**Figure 4:**
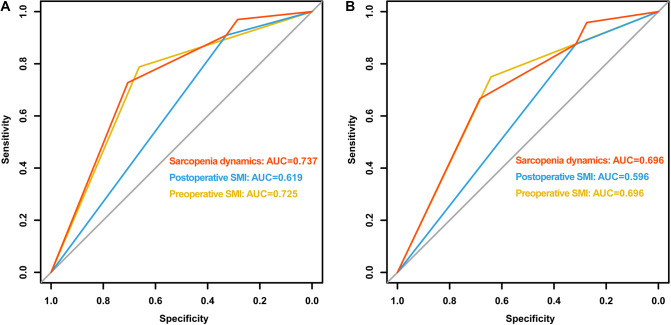
Comparison of area under ROC curves for preoperative SMI, postoperative SMI and sarcopenia dynamics. (**A**) OS ROC curves; (**B)** CSS ROC curves. Abbreviations: OS, overall survival; CSS, cancer-specific survival; ROC, receiver operator characteristic; AUC, area under the curve; SMI, skeletal muscle index.

Subsequently, we first used ROC curves to assess the predictive ability of preoperative SMI, postoperative SMI and sarcopenia dynamics on OS and CSS in RCC patients undergoing laparoscopic nephrectomy. The results showed that sarcopenia dynamics had a highest predictive ability for OS (AUC = 0.737, 95% CI 0.654–0.821, *p* < 0.001) compared with pre- and postoperative SMI, but was consistent with preoperative SMI for CSS (AUC = 0.696, 95% CI 0.593–0.798, *p* = 0.002) (Figure 4 and Table 3). In addition, multivariate Cox regression analysis showed that sarcopenia dynamics was an independent risk factor for OS and CSS, with a 73.6% reduction in OS risk (HR = 0.264, 95% CI 0.106–0.655, *p* = 0.004) and a 66.2% reduction in CSS risk (HR = 0.338, 95% CI 0.121–0.943, *p* = 0.038) in the group 2, and a 94.5% reduction in OS risk (HR = 0.055, 95% CI 0.007–0.407, *p* = 0.003) and a 91.9% reduction in CSS risk (HR = 0.081, 95% CI 0.011–0.616, *p* = 0.015) in the group 4 compared with the group 1 (Tables 4, 5).

**Table 3:** Analysis of predictive accuracy through the evaluation of the area under the curve (AUC).

Characteristic	Overall Survival			**Cancer-specific Survival**		
	AUC	**95% CI**	**P value**	AUC	**95% CI**	**P value**
Preoperative SMI	0.725	0.636–0.814	<0.001	0.696	0.589–0.803	0.002
Postoperative SMI	0.619	0.528–0.710	0.027	0.596	0.488–0.704	0.122
Sarcopenia dynamics	0.737	0.654–0.821	<0.001	0.696	0.593–0.798	0.002

*AUC, Area under the curve; CI, confidence interval; SMI, skeletal muscle index.*

**Table 4:** Univariate and multivariate analyses of factors associated with overall survival (OS).

Characteristics	Univariate analyses		Multivariate analyses	
	Hazard Ratio (95% CI)	*P* value	Hazard Ratio (95% CI)	*P* value
Age categorized, y				** **
≤65	Reference		Reference	
>65	2.225 (1.115–4.439)	0.023	-	0.621
Sex				
Male	Reference			
Female	0.915 (0.435–1.923)	0.814		
BMI categorized, kg/m^2^				
<25	Reference			
≥25	0.656 (0.323–1.333)	0.244		
Hypertension				
No	Reference			
Yes	1.113 (0.561–2.210)	0.759		
Diabetes				
No	Reference			
Yes	0.725 (0.255–2.063)	0.547		
Cardiovascular diseases				
No	Reference			
Yes	1.298 (0.501–3.365)	0.592		
Smoking				
No	Reference			
Yes	0.847 (0.327–2.193)	0.732		
Surgery type				
Partial nephrectomy	Reference		Reference	
Radical nephrectomy	2.824 (1.368–5.831)	0.005	-	0.556
Laterality				
Right	Reference			
Left	1.076 (0.542–2.135)	0.834		
AJCC stage				
I	Reference		Reference	
II	1.365 (0.315–5.921)	0.677	-	0.970
III	2.509 (0.987–6.374)	0.053	-	0.063
IV	6.025 (2.599–13.969)	<0.001	-	0.668
T-stage				
T1	Reference		Reference	
T2	1.866 (0.545–6.385)	0.320	-	0.579
T3	4.338 (2.026–9.290)	<0.001	-	0.084
T4	2.758 (0.637–11.953)	0.175	-	0.848
N-stage				
N0	Reference		Reference	
N1	4.565 (1.599–13.031)	0.005	-	0.420
M-stage				
M0	Reference		Reference	
M1	7.132 (3.087–16.480)	<0.001	5.305 (2.265–12.425)	<0.001
Fuhrman grade				
I	Reference		Reference	
II	3.298 (0.774–14.047)	0.107	-	0.926
III	4.749 (1.005–22.450)	0.049	-	0.660
IV	14.240 (1.248–162.495)	0.033	-	0.109
Sarcopenia dynamics				
Group 1	Reference		Reference	
Group 2	0.224 (0.092–0.550)	0.001	0.264 (0.106–0.655)	0.004
Group 3	0.491 (0.116–2.086)	0.335	0.392 (0.090–1.706)	0.212
Group 4	0.049 (0.007–0.365)	0.001	0.055 (0.007–0.407)	0.003

*OS, Overall survival; CI, confidence interval; BMI, Body mass index; AJCC, American Joint Committee on Cancerqi.*

**Table 5:** Univariate and multivariate analyses of factors associated with and cancer-specific survival (CSS).

Characteristics	Univariate analyses		Multivariate analyses	
	Hazard Ratio (95% CI)	*P* value	Hazard Ratio (95% CI)	*P* value
Age categorized, y				** **
≤65	Reference			
>65	1.249 (9.518–3.012)	0.621		
Sex				
Male	Reference			
Female	0.695 (0.276–1.751)	0.440		
BMI categorized, kg/m^2^				
<25	Reference			
≥25	0.827 (0.367–1.863)	0.647		
Hypertension				
No	Reference			
Yes	1.876 (0.832–4.227)	0.129		
Diabetes				
No	Reference			
Yes	0.750 (0.224–2.515)	0.641		
Cardiovascular diseases				
No	Reference			
Yes	0.667 (0.157–2.838)	0.583		
Smoking				
No	Reference			
Yes	0.430 (0.101–1.827)	0.253		
Surgery type				
Partial nephrectomy	Reference		Reference	
Radical nephrectomy	3.479 (1.441–8.400)	0.006	-	0.349
Laterality				
Right	Reference			
Left	1.060 (0.475–2.367)	0.887		
AJCC stage				
I	Reference		Reference	
II	1.875 (0.418–8.400)	0.412	-	0.658
III	1.808 (0.509–6.421)	0.360	-	0.449
IV	7.501 (2.951–19.067)	<0.001	-	0.451
T-stage				
T1	Reference		Reference	
T2	2.553 (0.717–9.085)	0.148	-	0.255
T3	3.999 (1.568–10.196)	0.004	-	0.554
T4	3.870 (0.865–17.323)	0.077	-	0.988
N-stage				
N0	Reference		Reference	
N1	6.655 (2.262–19.578)	0.001	-	0.163
M-stage				
M0	Reference		Reference	
M1	8.946 (3.565–22.641)	<0.001	5.305 (2.265–12.425)	<0.001
Fuhrman grade				
I	Reference		Reference	
II	4.603 (0.607–34.911)	0.140	-	0.678
III	9.803 (1.221–78.742)	0.032	-	0.157
IV	-		-	0.697
Sarcopenia dynamics				
Group 1	Reference		Reference	
Group 2	0.275 (0.100–0.752)	0.012	0.338 (0.121–0.943)	0.038
Group 3	0.722 (0.165–3.157)	0.665	0.521 (0.114–2.385)	0.400
Group 4	0.072 (0.009–0.543)	0.011	0.081 (0.011–0.616)	0.015

*CSS, Cancer-specific survival; CI, confidence interval; BMI, Body mass index; AJCC, American Joint Committee on Cancer.*

## Discussion

To our knowledge, this is the first report examining the prognostic survival outcomes of patients undergoing laparoscopic nephrectomy for RCC in relation to the pattern of change in sarcopenia. Our study found that the prevalence of postoperative sarcopenia assessed by SMI increased significantly compared to preoperative sarcopenia prevalence, from 39.5% to 70.1%. The prognostic survival of patients with pre- or postoperative SMI-assessed sarcopenia was worse than that of non-sarcopenia patients. More importantly, patients with group 4 sarcopenia dynamic (both pre- and postoperative non-sarcopenia) and patients with group 2 sarcopenia dynamic (preoperative non-sarcopenia to postoperative sarcopenia) had significantly better survival than the other two groups. After adjusting for other clinical factors, sarcopenia dynamics remained a prognostically independent risk factor for OS and CSS.

Sarcopenia is a syndrome characterized by progressive, widespread reduction in skeletal muscle content and hypofunction, and was first proposed by Rosenberg in 1989 (19). According to a German statistical report, the prevalence of sarcopenia ranges from 5% to 13% in older adults aged 60–70 years and from 11% to 50% in those aged 80 years or older (20). In our study, we found that the prevalence of preoperative sarcopenia was 56.3% in those >65 years of age, while the prevalence of postoperative sarcopenia increased to 87.5%. Patients with sarcopenia are more likely to experience cognitive impairment, frailty, decreased physical function, and significantly increased incidence of falls, fractures, and disability (21, 22). It is evident that sarcopenia has a high prevalence in the elderly population and poses a serious health risk to the elderly.

The relevance of sarcopenia to patients with RCC has received increasing attention in recent years. Patients with RCC often experience nausea, vomiting, and loss of appetite due to the disease itself or targeted therapy, resulting in reduced dietary intake. Psutka et al. (23) reviewed clinical data from 387 patients with localized RCC who underwent radical nephrectomy between 2000 and 2010 and found that 180 (47%) had sarcopenia. Ishihara et al. (24) investigated 71 patients with metastatic RCC treated with sunitinib in Japan from 2007 to 2014 and found that 45 (63.4%) patients had sarcopenia. In addition, Fukushima et al. (25) retrospectively studied data from 92 patients with metastatic RCC between 2003 and 2014 and found that up to 68% of patients were classified as having sarcopenia. In the present study, the percentage of preoperative sarcopenia was 39.5% and increased to 70.1% in the postoperative period, which could be attributed to a combination of perioperative fasting, postoperative stress, and intestinal inflammation, resulting in weight loss and increased risk of malnutrition in patients.

Several previous studies have reported preoperative sarcopenia as a valuable prognostic factor in patients with RCC. By retrospectively analyzing 632 patients with pT1-2 RCC who underwent radical nephrectomy from 2004 to 2014, Lee et al. (26) found that sarcopenia at diagnosis was an independent risk factor for postoperative all-cause and cancer-specific mortality in patients with pT1-2 RCC. Higgins et al. (27) found that sarcopenia was independently associated with poorer OS, CSS and recurrence-free survival (RFS) and that sarcopenia was associated with an increased likelihood of recurrence and death. A meta-analysis showed that patients with sarcopenia had worse OS (HR = 1.76; 95% CI, 1.35–2.31; *P* < 0.001). In addition, our previous studies confirmed that sarcopenia was an independent prognostic risk factor for patients with RCC (16, 17, 28).

However, previous studies have focused more on preoperative SMI diagnosed sarcopenia, whereas the prognostic role of postoperative SMI assessed sarcopenia and the dynamics of sarcopenia were unclear. Due to the invasiveness of surgery, some major operations can have a significant impact on the physical condition of patients, and some patients may experience muscle loss after surgery (29). In addition, postoperative related targeted therapy may lead to an associated gastrointestinal inflammatory response, which may have an impact on patient feeding and intestinal function, resulting in reduced intake and impaired nutrient absorption, and ultimately patient weight loss and increased risk of malnutrition (30). In this study, we present for the first time the prognostic value of sarcopenia as assessed by postoperative SMI and the dynamics of sarcopenia in patients undergoing laparoscopic nephrectomy for RCC. We found that patients with a diagnosis of sarcopenia both pre- and postoperatively had the worst prognostic status, and that a diagnosis of non-sarcopenia either pre- or postoperatively improved the prognosis of patients with RCC.

There are several shortcomings in this study: this study is a single-center retrospective study design, and only muscle mass to define sarcopenia, which is a limitation of this study. In addition, patient-related information in this study was collected from the patients’ inpatient medical records, and it is inevitable that some information was not recorded accurately, which may be subject to some selection bias and recall bias. Moreover, we did not follow up patients’ chemotherapy information, nutritional status and surgical complication information due to the incompleteness of the data. Finally, considering that our study is a single-institution retrospective study, we expect that a multicenter, large-scale prospective study can be conducted in the future to further validate the correlation between sarcopenia dynamics and prognosis of RCC patients.

## Conclusion

In conclusion, our study showed that the dynamics of sarcopenia between pre- and postoperative status was identified as a significant predictor of survival outcome in RCC patients undergoing laparoscopic nephrectomy and that maintaining a better nutritional status preoperatively and postoperatively is essential for long-term survival of RCC patients.

## Data Availability

The original contributions presented in the study are included in the article/supplementary materials, further inquiries can be directed to the corresponding author/s.

## References

[1] Hsieh JJ Purdue MP Signoretti S Swanton C Albiges L Schmidinger M Renal cell carcinoma. Nat Rev Dis Primers. (2017) 3:17009. 10.1038/nrdp.2017.9 28276433 PMC5936048

[2] Sung H Ferlay J Siegel RL Laversanne M Soerjomataram I Jemal A Global Cancer Statistics 2020: GLOBOCAN estimates of incidence and mortality worldwide for 36 Cancers in 185 Countries. CA Cancer J Clin. (2021) 71(3):209–49. 10.3322/caac.21660 33538338

[3] Mao W Wang K Xu B Zhang H Sun S Hu Q ciRS-7 is a prognostic biomarker and potential gene therapy target for renal cell carcinoma. Mol Cancer. (2021) 20(1):142. 10.1186/s12943-021-01443-2 34740354 PMC8570002

[4] Kotecha RR Motzer RJ Voss MH. Towards individualized therapy for metastatic renal cell carcinoma. Nat Rev Clin Oncol. (2019) 16(10):621–33. 10.1038/s41571-019-0209-1 30992569

[5] Tannir NM Pal SK Atkins MB. Second-line treatment landscape for renal cell carcinoma: a comprehensive review. Oncologist. (2018) 23(5):540–55. 10.1634/theoncologist.2017-0534 29487224 PMC5947457

[6] Yong C Stewart GD Frezza C. Oncometabolites in renal cancer. Nat Rev Nephrol. (2020) 16(3):156–72. 10.1038/s41581-019-0210-z 31636445 PMC7030949

[7] Lorenzi M Bonassi S Lorenzi T Giovannini S Bernabei R Onder G. A review of telomere length in sarcopenia and frailty. Biogerontology. (2018) 19(3–4):209–21. 10.1007/s10522-018-9749-5 29549539

[8] Blum D Omlin A Baracos VE Solheim TS Tan BH Stone P Cancer cachexia: a systematic literature review of items and domains associated with involuntary weight loss in cancer. Crit Rev Oncol Hematol. (2011) 80(1):114–44. 10.1016/j.critrevonc.2010.10.004 21216616

[9] GiovanniniSOnderGLattanzioFBustacchiniSDi StefanoGMoresiR Selenium concentrations and mortality among community-dwelling older adults: results from IlSIRENTE study. J Nutr Health Aging. (2018) 22(5):608–12. 10.1007/s12603-018-1021-929717761

[10] Giovannini S Onder G Leeuwenburgh C Carter C Marzetti E Russo A Myeloperoxidase levels and mortality in frail community-living elderly individuals. J Gerontol A Biol Sci Med Sci. (2010) 65(4):369–76. 10.1093/gerona/glp183 20064836 PMC4345789

[11] Cruz-Jentoft AJ Bahat G Bauer J Boirie Y Bruyere O Cederholm T Sarcopenia: revised European consensus on definition and diagnosis. Age Ageing. (2019) 48(1):16–31. 10.1093/ageing/afy169 30312372 PMC6322506

[12] Bauer J Morley JE Schols A Ferrucci L Cruz-Jentoft AJ Dent E Sarcopenia: a time for action. An SCWD position paper. J Cachexia Sarcopenia Muscle. (2019) 10(5):956–61. 10.1002/jcsm.12483 31523937 PMC6818450

[13] Cruz-Jentoft AJ Baeyens JP Bauer JM Boirie Y Cederholm T Landi F Sarcopenia: European consensus on definition and diagnosis: report of the European Working Group on Sarcopenia in Older People. Age Ageing. (2010) 39(4):412–23. 10.1093/ageing/afq034 20392703 PMC2886201

[14] Caan BJ Cespedes Feliciano EM Prado CM Alexeeff S Kroenke CH Bradshaw P Association of muscle and adiposity measured by computed tomography with survival in patients with nonmetastatic breast cancer. JAMA Oncol. (2018) 4(6):798–804. 10.1001/jamaoncol.2018.0137 29621380 PMC6584322

[15] Kobayashi A Kaido T Hamaguchi Y Okumura S Shirai H Yao S Impact of sarcopenic obesity on outcomes in patients undergoing hepatectomy for hepatocellular carcinoma. Ann Surg. (2019) 269(5):924–31. 10.1097/SLA.0000000000002555 29064889

[16] Mao W Wang K Zhang H Lu H Sun S Tian C Sarcopenia as a poor prognostic indicator for renal cell carcinoma patients undergoing nephrectomy in China: a multicenter study. Clin Transl Med. (2021) 11(1):e270. 10.1002/ctm2.270 33463055 PMC7775986

[17] Mao W Zhang N Wang K Hu Q Sun S Xu Z Combination of albumin-globulin score and sarcopenia to predict prognosis in patients with renal cell carcinoma undergoing laparoscopic nephrectomy. Front Nutr. (2021) 8:731466. 10.3389/fnut.2021.731466 34631767 PMC8495413

[18] Kang M Chang CT Sung HH Jeon HG Jeong BC Seo SI Prognostic significance of pre- to postoperative dynamics of the prognostic nutritional index for patients with renal cell carcinoma who underwent radical nephrectomy. Ann Surg Oncol. (2017) 24(13):4067–75. 10.1245/s10434-017-6065-2 28975525

[19] Rosenberg I. Epidemiologic and methodologic problems in determining nutritional status of older persons. Proceedings of a conference. Albuquerque, New Mexico, October 19–21, 1988. Am J Clin Nutr. (1989) 50(5 Suppl):1121–35. 2816807

[20] von Haehling S Morley JE Anker SD. An overview of sarcopenia: facts and numbers on prevalence and clinical impact. J Cachexia Sarcopenia Muscle. (2010) 1(2):129–33. 10.1007/s13539-010-0014-2 21475695 PMC3060646

[21] Beaudart C Reginster JY Petermans J Gillain S Quabron A Locquet M Quality of life and physical components linked to sarcopenia: the SarcoPhAge study. Exp Gerontol. (2015) 69:103–10. 10.1016/j.exger.2015.05.003 25979160

[22] Scott D Hayes A Sanders KM Aitken D Ebeling PR Jones G. Operational definitions of sarcopenia and their associations with 5-year changes in falls risk in community-dwelling middle-aged and older adults. Osteoporosis Int. (2014) 25(1):187–93. 10.1007/s00198-013-2431-5 23800748

[23] Psutka SP Boorjian SA Moynagh MR Schmit GD Costello BA Thompson RH Decreased skeletal muscle mass is associated with an increased risk of mortality after radical nephrectomy for localized renal cell cancer. J Urol. (2016) 195(2):270–6. 10.1016/j.juro.2015.08.072 26292038

[24] Ishihara H Kondo T Omae K Takagi T Iizuka J Kobayashi H Sarcopenia and the modified glasgow prognostic score are significant predictors of survival among patients with metastatic renal cell carcinoma who are receiving first-line sunitinib treatment. Target Oncol. (2016) 11(5): 605–17. 10.1007/s11523-016-0430-0 27023922

[25] Fukushima H Nakanishi Y Kataoka M Tobisu K Koga F. Prognostic significance of sarcopenia in patients with metastatic renal cell carcinoma. J Urol. (2016) 195(1):26–32. 10.1016/j.juro.2015.08.071 26292042

[26] Lee J Suh J Song C You D Jeong IG Hong B Association between sarcopenia and survival of patients with organ-confined renal cell carcinoma after radical nephrectomy. Ann Surg Oncol. (2021) 29(4):2473-79. 10.1245/s10434-021-10881-7 34625877

[27] Higgins MI Martini DJ Patil DH Nabavizadeh R Steele S Williams M Sarcopenia and modified Glasgow Prognostic Score predict postsurgical outcomes in localized renal cell carcinoma. Cancer. (2021) 127(12): 1974–83. 10.1002/cncr.33462 33760232

[28] Hu Q Mao W Wu T Xu Z Yu J Wang C High neutrophil-to-lymphocyte ratio and platelet-to-lymphocyte ratio are associated with sarcopenia risk in hospitalized renal cell carcinoma patients. Front Oncol. (2021) 11:736640. 10.3389/fonc.2021.736640 34760698 PMC8573165

[29] van Venrooij LM Verberne HJ de Vos R Borgmeijer-Hoelen MM van Leeuwen PA de Mol BA. Postoperative loss of skeletal muscle mass, complications and quality of life in patients undergoing cardiac surgery. Nutrition. (2012) 28(1):40–5. 10.1016/j.nut.2011.02.007 21621393

[30] Loriot Y Perlemuter G Malka D Penault-Llorca F Boige V Deutsch E Drug insight: gastrointestinal and hepatic adverse effects of molecular-targeted agents in cancer therapy. Nat Clin Pract Oncol. (2008) 5(5): 268–78. 10.1038/ncponc1087 18349858

